# Image Monitoring of Pharmaceutical Blending Processes and the Determination of an End Point by Using a Portable Near-Infrared Imaging Device Based on a Polychromator-Type Near-Infrared Spectrometer with a High-speed and High-Resolution Photo Diode Array Detector

**DOI:** 10.3390/molecules20034007

**Published:** 2015-03-03

**Authors:** Kodai Murayama, Daitaro Ishikawa, Takuma Genkawa, Hiroyuki Sugino, Makoto Komiyama, Yukihiro Ozaki

**Affiliations:** 1 Innovation Headquarters, Yokogawa Electric Corporation, 2-9-32 Nakacho, Musashino, Tokyo 180-8750, Japan; E-Mails: H.Sugino@jp.yokogawa.com (H.S.); Makoto.Komiyama@jp.yokogawa.com (M.K.); 2 Graduate School of Agricultural Science, Tohoku University, 1-1 Amamiya, Tsutsumidori, Aobaku, Sendai 981-8555, Japan; E-Mail: daitaroishikawa@m.tohoku.ac.jp; 3 School of Science and Technology, Kwansei Gakuin University, 2-1 Gakuen, Sanda, Hyogo 669-1337, Japan; 4 Faculty of Life and Environmental Sciences, University of Tsukuba, 1-1-1 Tennodai, Tsukuba, Ibaraki 305-8572, Japan; E-Mail: genkawa.takuma.fm@u.tsukuba.ac.jp

**Keywords:** near-infrared imaging, powder blending, in-line monitoring

## Abstract

In the present study we have developed a new version (ND-NIRs) of a polychromator-type near-infrared (NIR) spectrometer with a high-resolution photo diode array detector, which we built before (D-NIRs). The new version has four 5 W halogen lamps compared with the three lamps for the older version. The new version also has a condenser lens with a shorter focal point length. The increase in the number of the lamps and the shortening of the focal point of the condenser lens realize high signal-to-noise ratio and high-speed NIR imaging measurement. By using the ND-NIRs we carried out the in-line monitoring of pharmaceutical blending and determined an end point of the blending process. Moreover, to determinate a more accurate end point, a NIR image of the blending sample was acquired by means of a portable NIR imaging device based on ND-NIRs. The imaging result has demonstrated that the mixing time of 8 min is enough for homogeneous mixing. In this way the present study has demonstrated that ND-NIRs and the imaging system based on a ND-NIRs hold considerable promise for process analysis.

## 1. Introduction

In modern pharmaceutical industries, the idea of new quality management has been proposed for the delivery of reliable pharmaceutical products. The idea is the quality by design (QbD) approach. QbD is an approach that clarifies the factors which significantly influence the quality properties of the medicine from the stage of the formulation design and the process design, and it carries out the quality control based on a scientific basis [1]. Process analytical technology (PAT) is used to analyze the manufacturing process, determine the factors essential for understanding the manufacturing process, and clarify variable factors that affect quality control [2,3]. PAT is positioned as a powerful technology to achieve the QbD approach. To evaluate the quality of an oral medication, the monitoring of component distribution including that of active pharmaceutical ingredient (API) is essential. It is well known that spectroscopic technology is attractive for the non-destructive monitoring of pharmaceutical tablets [3,4,5,6,7,8,9,10,11]. Evaluation methods for inhomogeneity in a tablet based on ultraviolet-visible (UV-Vis) spectroscopy and high performance liquid chromatography (HPLC) were developed about two decades ago [10,11]. However, the UV-Vis spectroscopy methods are usually based on destructive methods, so there are only a few reports on the use of UV-Vis spectroscopy in the quantitative analysis of tablet components. Recently a number of research groups have been involved in the development of vibrational spectroscopy methods for PAT [4,5,6,7,8,9,10]. Near-infrared (NIR) spectroscopy in particular has attracted keen interest as a powerful PAT method since it has superior features for real time monitoring; it is a non-destructive and non-contact method [4,12]. Moreover, NIR can use optical fibers, allowing remote-sensing. Imaging techniques in the NIR region have also been developing [13,14,15,16,17,18,19].

Our research group has been involved in PAT studies through the development of new NIR instruments, particularly, imaging instruments [15,19,20,21]. We recently developed a distribution type NIR spectrometer (D-NIRs), which is an NIR imaging instrument based on a polychromator-type NIR spectrometer with a high sensitivity and high-resolution photodiode array detector (PDA). The portability of D-NIRs is superior. The size of the imaging unit is only 3 liter volume. D-NIRs enables high speed monitoring (25 ms/pixel) and high wavelength resolution (1.25 nm) of NIR imaging due to the newly developed Indium Gallium Arsenide (InGaAs) detector. The detector consists of a high-density PDA containing 640 elements with 20-μm pitch. InGaAs photodiodes with a wavelength sensitivity of 900–1700 nm (photoreceptive sensitivity: 0.8 at 1550 nm) are used [20].

A NIR image obtained during the water dissolution process of a tablet demonstrated the potential of D-NIRs as a PAT tool [19,22,23,24]. Of course, NIR images of tablets measured by using D-NIRs can clarify the distribution and the relative abundance of chemical components in the tablets [15]. It was successfully demonstrated that D-NIRs is an attractive tool for PAT and that it can be applied for QbD. 

However, evaluation methods for pharmaceutical blending process based on NIR imaging are still under development, although the blending process can be monitored by NIR spectra [10,11,25] and it was reported that NIR imaging technique can be utilized for studying the effects of the particle sizes of chemical components on the blending process [26]. In addition, the end point of the blending process was determined by using a NIR imaging technique [27]. However, in this case it took long time to get an image and also the wavelength resolution was very low. Thus, it would be highly desirable to develop a NIR imaging system that enables both high speed and high wavelength resolution measurements in NIR imaging.

Quite recently, as a part of the present study we have built a new type of D-NIRs named ND-NIRs. The new type has four 5 W halogen lamps as light sources, that is, one more halogen lamp than the original type. In addition, in the scanning unit of ND-NIRs the focal length of the condenser lens is shortened to optimize the performance of the optical scanning system. The increase in the number of lamps together with the shortening of the focal length has enabled about seven times faster imaging measurements compared with the original version. The purpose of the present study was to carry out the in-line monitoring of a pharmaceutical blending process and the determination of an end point of this blending process by using a ND-NIRs, polychromator-type NIR spectrometer with high speed and high wavelength resolution. Moreover, to determinate an accurate end point in a short time, a NIR image of the blending sample was acquired by means of a portable NIR imaging device based on ND-NIRs.

## 2. Results and Discussion

### 2.1. NIR Spectra of Ascorbic Acid, D-mannitol and Magnesium Stearate

**Figure 1:**
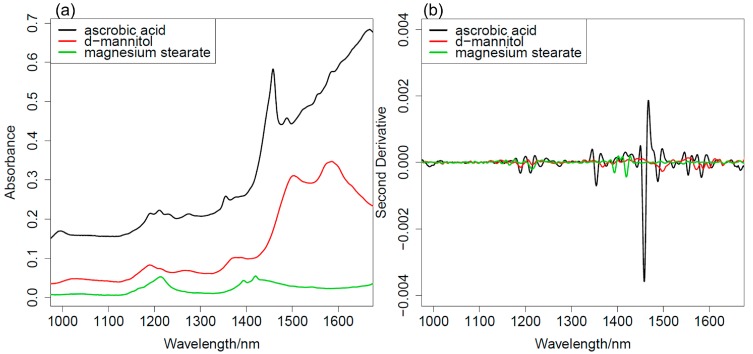
(**a**) NIR spectra of ascorbic acid, D-mannitol and magnesium stearate; (**b**) The second derivative of the spectra shown in (a).

Figure 1 shows: (a) diffuse-reflectance (DR) NIR spectra and (b) the second derivative spectra of powder of ascorbic acid (AsA), D-mannitol and magnesium stearate in the 950–1700 nm region. Narrow bands of AsA at around 1361 and 1458 nm may be due to the first overtones of stretching vibrations of free and intermolecular hydrogen-bonded OH groups, respectively [28]. A broad absorption in the 1150–1250 nm region of AsA may be assigned to the second overtones of CH stretching vibrations [28]. A broad absorption in the 1490–1590 nm region of D-mannitol may be assigned to the first overtones of OH stretching vibrations.

### 2.2. Evaluation of Inhomogeneity during Blending Process by In-Line NIR Spectrometer

**Figure 2:**
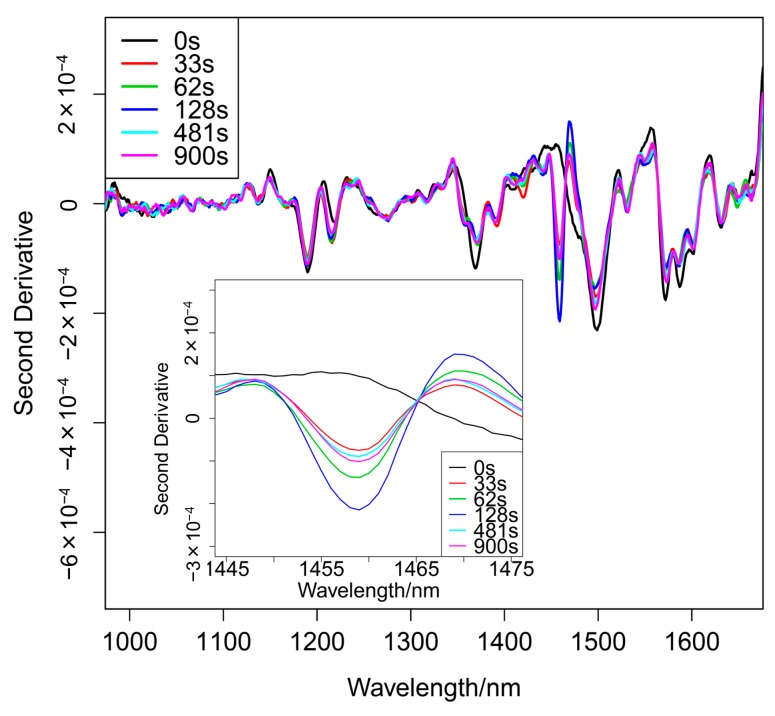
The second derivative spectra of mixing sample measured during the blending process.

Figure 2 shows a series of second derivative spectra of the blending sample measured during rotation mixing. The inset of Figure 2 depicts the enlargement of the 1445–1475 nm region. It is noted that the peak intensity at 1458 nm due to the first overtone of the OH stretching vibration mode of AsA changes with time. The band of 1458 nm is not detected in the initial stage of the mixing. However, this band becomes strong as mixing proceeds. The spectrum measured at 0 s reflects D-mannitol only; the band at 1495 nm arises only from the first overtone of the OH stretching mode. The intensity at 1495 nm becomes intense with mixing.

**Figure 3:**
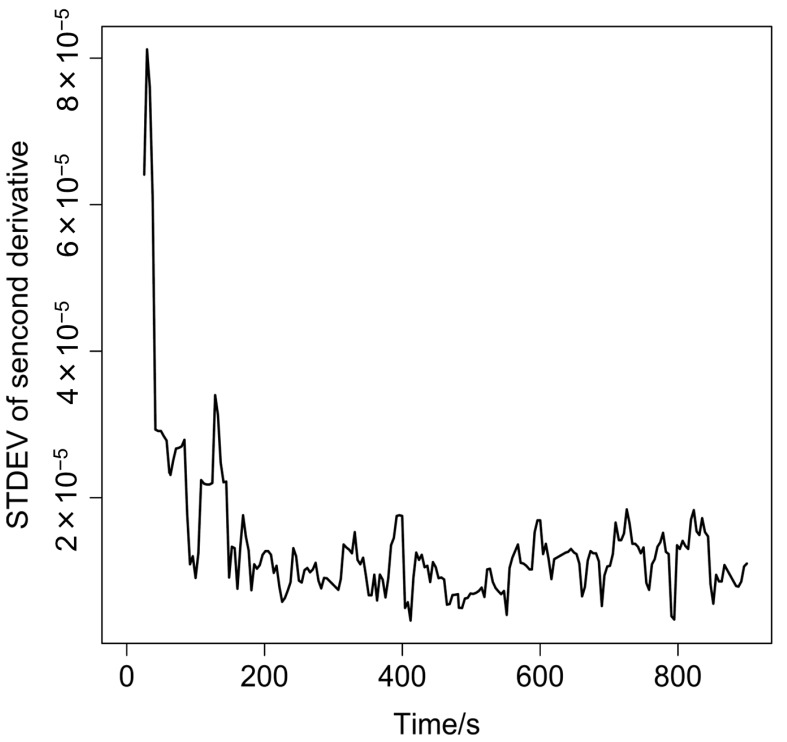
A plot for standard deviation of the second derivative intensity at 1,458 nm *versus* time. The DR-NIR spectrum was measured with 4 s intervals.

Figure 3 plots a 5-point moving block standard deviation of the peak intensity at 1458 nm in the second-derivative spectra *versus* time. We also prepared similar plot by using the band at 1495 nm due to D-mannitol. The 1495 nm band gave similar result to the 1458 nm band. It is noted in Figure 3 that during the initial stage of mixing, the powder sample in the vessel is inhomogeneous, and therefore, the standard deviation (SD) value is high. As mixing proceeds, the sample becomes homogeneous. Then, the spectra of the mixtures of ascorbic acid, D-mannitol and magnesium stearate as the mixing ratio are detected, and thus, the spectral variation decreases, yielding a low SD value. The result in Figure 3 shows that the SD gives a constant value around 168 s after the start of rotation mixing. Thus, it is very likely that the sample in the bottle becomes homogeneous after 168 s. Note that the SD does not exceed 0.00002 after 168 s.

### 2.3. Evaluation of Inhomogeneity during Blending Process by NIR Imaging

**Figure 4:**
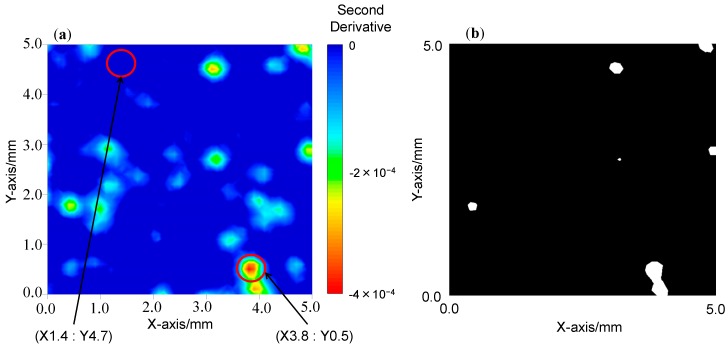
(**a**) The second derivative intensity mapping image of the mixing sample at 1 min after the start of the blending. (**b**) A binary image of the sample at 1 min after the start of the blending. A binary image developed by arbitrary threshold value.

**Figure 5:**
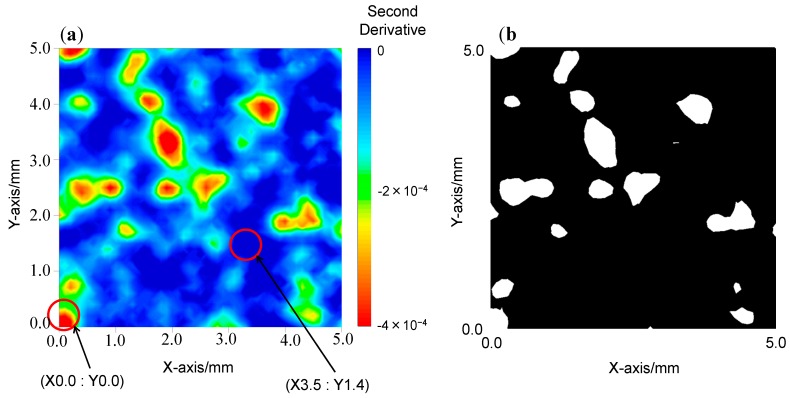
(**a**) The second derivative intensity mapping image of the mixing sample at 8 min after the start of the blending. (**b**) A binary image of the sample at 8 min after the start of the blending. A binary image developed by arbitrary threshold value.

**Figure 6:**
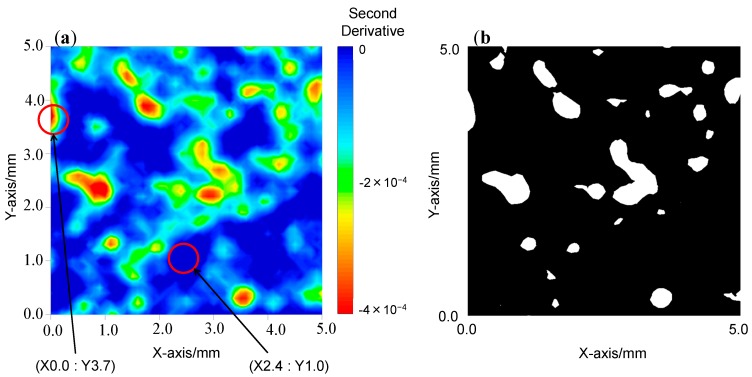
(**a**) The second derivative intensity mapping image of the mixing sample at 15 min after the start of the blending. (**b**) A binary image of the sample at 15 min after the start of the blending. A binary image developed by arbitrary threshold value.

**Figure 7:**
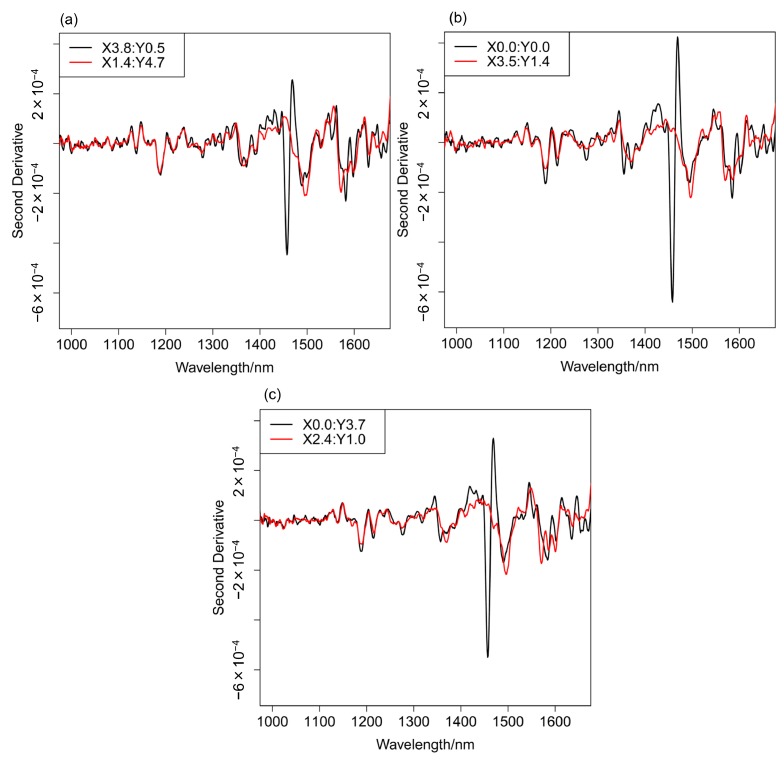
(**a**) The second derivative spectra at X3.8, Y0.5 and X1.4, Y4.7 points of the binary image of the blending at 1 min after the start of the blending. (**b**) Those at X0.0, Y0.0 and X3.5, Y1.4 points. (**c**) Those at X0.0, Y3.7 and X2.4, Y1.0 points.

Figure 4, Figure 5 and Figure 6 depict: (a) images for the distribution of AsA and (b) binary images for mixing times of 1, 8, and 15 min. A binary image is the two color image, it usually consists of white and black color. To determine the color in an image, the arbitrary threshold is defined prior to the visualization from the digital numbers and intensities, *etc.* of all pixels. Figure 7a–c show the second derivative spectra collected at different points in the two-dimensional image of the blend sample at 1 min after the start of blending. In the measurement of two-dimensional image of blend sample NIR spectra acquisition, second differentiation treatment, and absorbance mapping using specific wavelength were carried out at the same time. The ND-NIRs allows the spectral acquisition in 242 s for a measurement area of 5 mm × 5 mm, spatial resolution 0.1 mm in the wavelength region of 934–1713 nm, with the wavelength resolution of 1 nm. In this system the number of data is 2601 pixels times 780 absorbance data = 202,878 datapoints. Table 1 summarizes the measurement times for various measurement conditions. The results in the table demonstrate that ND-NIRs can provide two-dimensional spectra imaging data at a high speed. Second derivative spectra of one pixel (one point) shown in Figure 7 demonstrate that they catch clearly the specific absorption wavelength of 1458 nm due to AsA. These results suggest that ND-NIRs has high signal-to-noise ratio and high wavelength resolution, enabling the correction of base line deviation and the band separations without the influence of the increase of noise induced by the second derivative treatment. Each point spectrum obtained from the binary images corresponds well to the spectrum of AsA, showing that the sample existing in the points is AsA.

**Table 1:** Measurement time for each condition by ND-NIRs.

Measurement Area	5 mm × 5 mm			
**Spatial Resolution**	1 mm	0.5 mm	0.2 mm	0.1 mm
**Number of Measurement Point (Number of Spectra Data)**	36	121	676	2061
**Measurement Time**	5 s	13 s	64 s	242 s

We calculated area ratios (pixel ratios) of AsA and non-AsA from the binary images, and then estimated the temporal change in the relative amount of AsA. The area ratio of AsA in the binary images of two-dimensional spectra at the mixing time of 1, 8, and 15 min was 1.49%, 9.57%, and 8.74%, respectively. The relative amount of AsA at the mixing time of 8 and 15 min was almost the same as that the initial sample throw amount (10%). On the other hand, the relative amount of AsA at the mixing time of 1 min was much smaller, indicating that the mixing is insufficient. From the present result we can conclude that the mixing time of 8 min is enough for homogeneous mixing.

## 3. Experimental Section 

### 3.1. New NIR Imaging Device (ND-NIRs)

**Figure 8:**
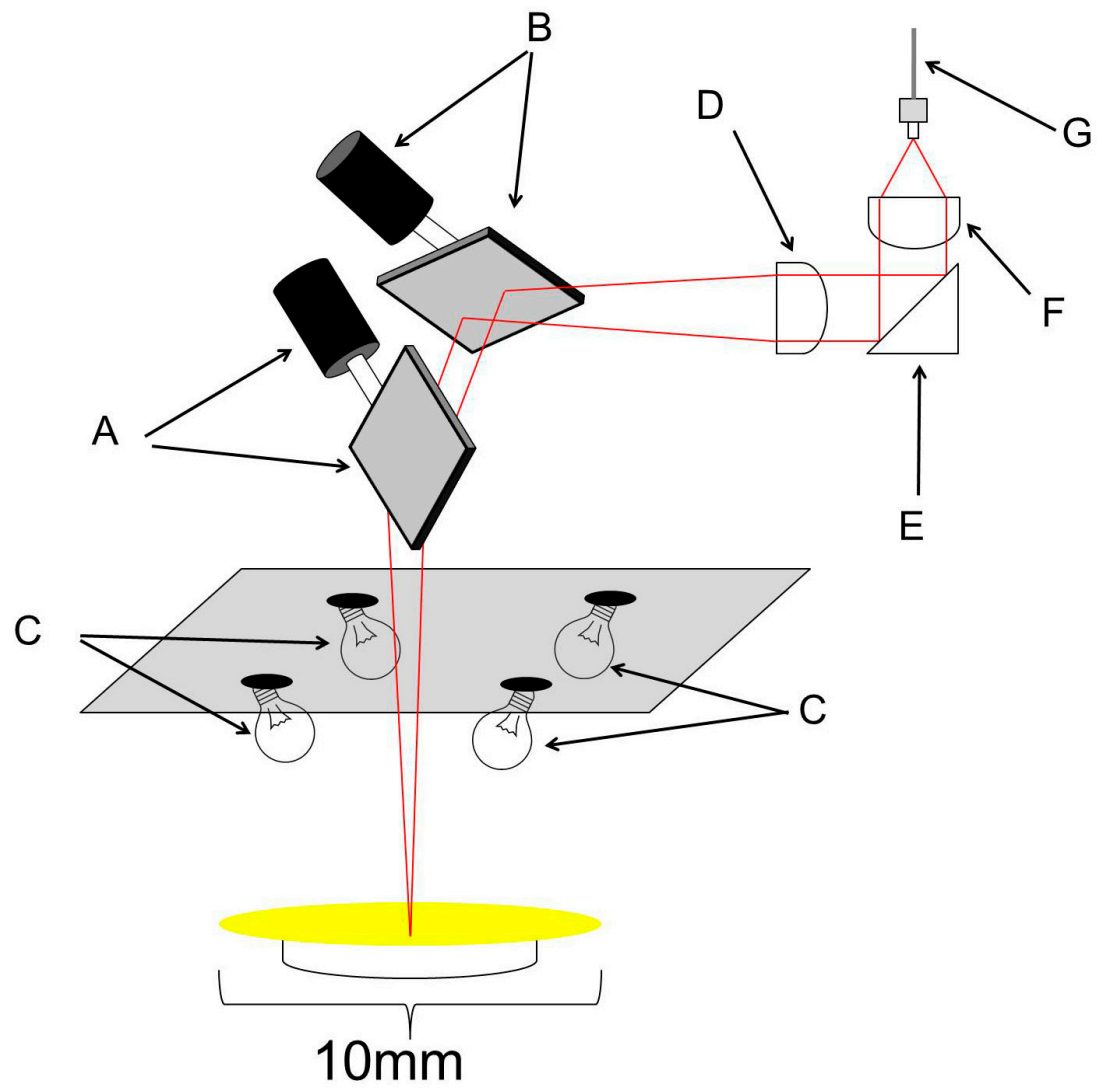
Overview of the developed NIR imaging device (ND-NIRs). A: X axis galvano mirror, B: Y axis galvano mirror, C: 5 W halogen lamp, D: Condensing lens 1, E: Right angle prism mirror, F: Condensing lens 2, G: Optical fiber cable.

**Figure 9:**
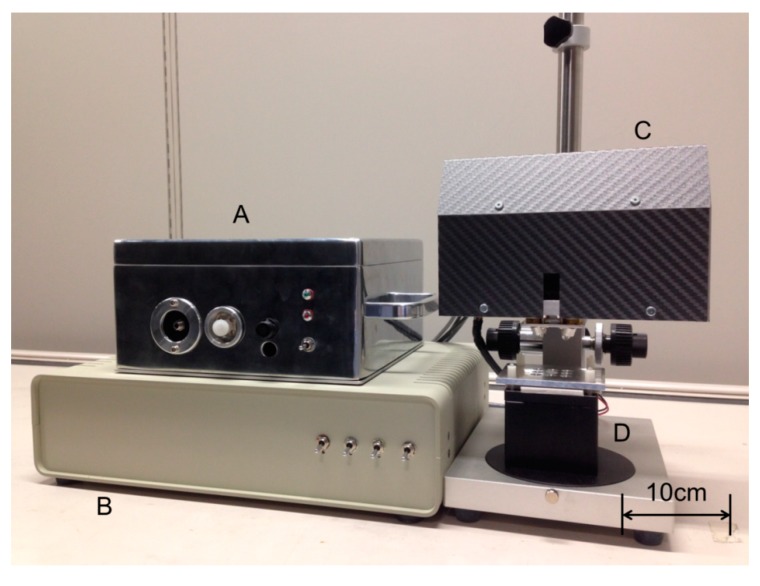
Outline of D-NIRs. A: P-NIRs, B: XY scanning unit, C: control unit, D: Sample plinth with temperature control function.

**Figure 10:**
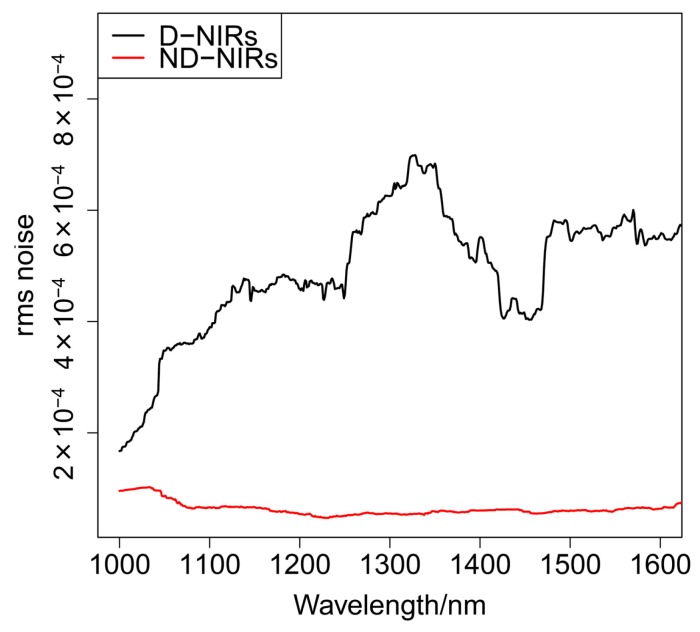
A plot for root mean square noise of D-NIRs and ND-NIRs at high-light flux.

A new version of D-NIRs (Yokogawa Electric Co., Tokyo, Japan) has been developed for the collection of two-dimensional DR-NIR spectra data. Its imaging unit consists of galvanic mirrors, condenser lenses, a rectangular prism, a fiber-optic cable, and four halogen lamps as shown in Figure 8 and Figure 9. There are two significant major differences between the original version (D-NIRs) and the new version (ND-NIRs). One is the number of lamps. The new version has four 5 W halogen lamps while the old version had only three. This increase in the number of lamps increased the light power significantly. Another important difference between the two versions is the length of the focal point of the condenser lens. The length has become significantly shorter in the new version (70 mm) compared with that in the old version (100 mm), greatly increasing the signal intensity. The increase in the number of the lamps and shortening of the focal length of the condenser lens achieve high signal-to-noise ratio, enabling high-speed NIR imaging measurement. Figure 10 plots a root mean square (rms) noise of D-NIRs and ND-NIRs at the high-light flux. The rms noise evaluation test between D-NIRs and ND-NIRs that was carried out in accordance with the US Pharmacopeia [29]. The rms noise level of the old version is 0.5 mAbs/0.5 s, while that of the new version is 0.05 mAbs/0.5 s. The measurement speed of the new version (11 s/100 pixels) has become seven times higher compared with that of the original one (80 s/100 pixels).

It is also notable that the size of the new imaging unit is only 220 × 90 × 150 mm due to the contribution of these optimized elements in the imaging unit. The irradiation energy from the four halogen lamps reaches a sample, and the diffuse reflected reaches the galvanic mirror. Finally, this signal is detected by a spectrometer named P-NIRs through the optical fiber. P-NIRs was also developed as a new spectrometer by our research group [20]. The high speed spectra mapping of 13 s per 25 mm^2^ area of ND-NIRs is due to the P-NIRs. The superior futures of P-NIRs were reported in a previous paper [20,21]. The maximum scan area of ND-NIRs is 10 × 10 mm, and its spatial resolution is 25 μm.

### 3.2. Data Collection

**Figure 11:**
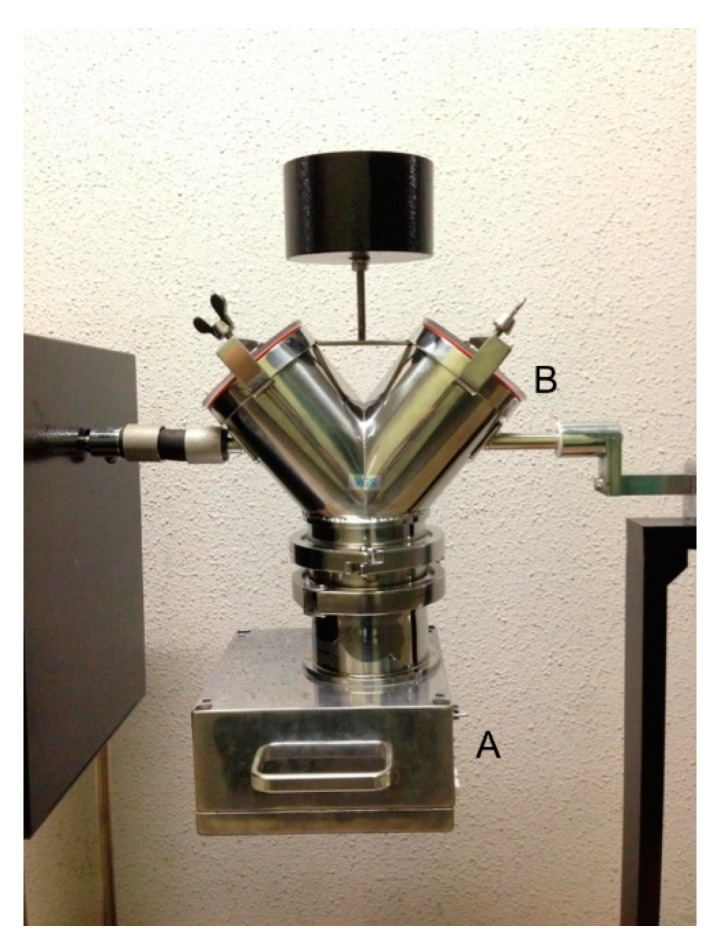
Outline of the blending test setup. A: P-NIRs for the in-line blending process monitor, B: Vessel-type blending machine.

The blending machine was rotated by 15 rpm in 15 min. The spectrometer, P-NIRs, was placed under this machine (Figure 11), and when the machine returned to the original position, the DR-NIR spectrum of blending powder was recorded by P-NIRs. The NIR spectra in the 950–1700 nm region were measured with a 1 nm interval, and they were converted to CSV format immediately. These spectral data were subjected to Savitzky-Golay (SG) smoothing and then to second derivative treatment with 19 point, and 2nd order [30]. Two-dimensional measurements of DR-NIR spectra were carried out several times by using a ND-NIRs. DR-NIR spectra at each pixel were obtained by line scanning method, and the peak at 1458 nm due to the first overtone of OH stretching vibration of AsA identified by the point measurement was used for the NIR mapping.

### 3.3. Sample Preparation

Ascorbic acid (AsA), magnesium stearate and D-mannitol were purchased from Kanto Chemical Co., Inc. (Tokyo, Japan), and their powders were prepared for blending. These materials were introduced into a vessel type blending machine (Tsutsui Scientific Instruments Co., Ltd., Taito-ku, Japan). The outline of the blending test setup is shown in Figure 3. To monitor the blending process, each material was added in the order D-mannitol, magnesium stearate and AsA. Finally, the prepared powder consisted of 88% (w/w) D-mannitol, 2% (w/w) magnesium stearate and 10% (w/w) AsA.

## 4. Conclusions

We have developed a new version of a polychromator-type NIR spectrometer with a high-resolution photo diode array detector, which we built before (D-NIRs). The new version has achieved a high signal-to-noise ratio and a high speed. The noise level of the new version is 10 times lower than that of the old version, and the measurement speed of the new version is seven times higher than that of the original one. By using the new version we performed an in-line evaluation of inhomogeneity during a blending process and determined the end point for homogeneous mixing. The evaluation of inhomogeneity during the blending process was also carried out by NIR imaging. Two-dimensional spectra of mixing samples were measured at different times. The NIR imaging revealed that the distribution of AsA in the blend sample changes with time. It has turned out that the mixing time of 8 min is sufficient for homogeneous mixing. The present study has demonstrated that NIR imaging is very useful for further understanding of the result of in-line monitoring of blending process.
